# The Value of Parental Karyotyping in Recurrent Pregnancy Loss Lies in Individual Risk Assessments

**DOI:** 10.3390/medicina60111778

**Published:** 2024-10-31

**Authors:** Gabriela Popescu-Hobeanu, Simona Serban Sosoi, Mihai Cucu, Ioana Streață, Amelia Dobrescu, Răzvan Pleșea, Anca Lelia Costache, Andreea Iordache, Bianca Petre-Mandache, Ștefania Tudorache, Alexandru Comănescu, Dominic Iliescu, Florin Burada

**Affiliations:** 1 Doctoral School, University of Medicine and Pharmacy of Craiova, 200349 Craiova, Romania; gmph94@gmail.com (G.P.-H.); i.andreea1506@gmail.com (A.I.); aknaib86@gmail.com (B.P.-M.); 2 Laboratory of Human Genomics, University of Medicine and Pharmacy of Craiova, 200638 Craiova, Romania; simona.sosoi@umfcv.ro (S.S.S.); ioana.streata@umfcv.ro (I.S.); amelia.dobrescu@umfcv.ro (A.D.); razvan.plesea@umfcv.ro (R.P.); anca.costache@umfcv.ro (A.L.C.); 3 Regional Centre of Medical Genetics Dolj, Emergency Clinical County Hospital Craiova, 200642 Craiova, Romania; 4 Department of Obstetrics and Gynecology, University of Medicine and Pharmacy of Craiova, 200349 Craiova, Romania; stefania.tudorache@gmail.com (Ș.T.); alexandru.comanescu@umfcv.ro (A.C.); dominic.iliescu@yahoo.com (D.I.); 5 Department of Obstetrics and Gynecology, Emergency Clinical County Hospital, 200642 Craiova, Romania

**Keywords:** recurrent pregnancy loss, conventional karyotyping, structural chromosome abnormalities, balanced translocation, Robertsonian translocation

## Abstract

*Background and Objectives:* Recurrent pregnancy loss (RPL) is a multifactorial condition, encompassing genetic, anatomical, immunological, endocrine, as well as infectious and environmental factors; however, the etiology remains elusive in a substantial number of cases. Genetic factors linked to RPL include parental karyotype abnormalities (e.g., translocations, inversions, copy number variants), an increase in sperm aneuploidy, fetal microchimerism, severe skewing of X chromosome inactivation, and various gene polymorphisms. Our study aims to explore the value of routine conventional parental karyotyping in couples with RPL. *Materials and Methods:* A total of 213 couples (426 individuals) with a history of RPL were enrolled in this retrospective study. The peripheral blood samples included in this study were referred to the Human Genomics Laboratory of the University of Medicine and Pharmacy in Craiova, Romania, for conventional cytogenetic analysis between January 2013 and December 2023, by the Outpatient Medical Genetics Clinic of the Emergency Clinical County Hospital of Craiova. Chromosome analysis was performed using standard protocols and karyotypes were reported according to ISCN. *Results:* Out of 426 patients provided with conventional G-banded chromosome analysis, 410 had a normal karyotype (96.2%) and 16 had chromosome abnormalities (3.8%). The most common chromosomal abnormalities were reciprocal and Robertsonian translocations, with chromosomes 8, 11, 14, and 21 being most frequently involved. A single numerical anomaly was detected (47,XYY). One or multiple chromosomal polymorphisms were identified in 104 subjects (24.4%). In addition, we conducted a stratified analysis of the unselected group and detected chromosome abnormalities in only four cases (0.94%). *Conclusions:* Our results are consistent with recommendations for paternal karyotyping after an individual risk assessment in instances such as a previous live birth with congenital anomalies and/or the detection of unbalanced chromosomes or a translocation in product of conception or chorionic villi/amniotic fluid samples. In the absence of a positive history, blindly karyotyping couples may prove too expensive and labor intensive, while providing no information on fertility status or live birth rates.

## 1. Introduction

The definition of recurrent pregnancy loss (RPL) has been a subject of debate amongst medical organizations for decades [1]. According to the Royal College of Obstetricians and Gynecologists (RCOG), RPL is defined as the loss of three or more consecutive pregnancies [2]. In contrast, the American Society for Reproductive Medicine (ASRM) describes RPL as two or more clinical (documented by ultrasonography or histopathology) miscarriages [3]. For this study, we aligned ourselves with the European Society of Human Reproduction and Embryology (ESHRE) guidelines, which state that a diagnosis of RPL could be considered after two pregnancy losses, from the moment of conception until 24 weeks of gestation, regardless of them being consecutive or not [4]. Given the variations in diagnostic criteria applied by various guidelines, the true global incidence of RPL is difficult to estimate. However, most studies place the overall incidence at 1–3% of couples attempting conception [5,6,7,8,9,10,11,12].

RPL is a multifactorial condition that can arise from a myriad of underlying causes, encompassing genetic, anatomical (septate uterus, intrauterine adhesions, submucosal uterine leiomyomas, polyps) [13], immunological (anti-phospholipid antibodies, antinuclear antibodies, anti-thyroid antibodies) [14,15,16], endocrine (polycystic ovarian syndrome, poorly controlled type I or II diabetes mellitus, untreated hypothyroidism, prolactin disorders) [17,18,19], as well as infectious and environmental factors (lifestyle factors, stress, or occupational exposure to chemicals or harmful materials) [20,21,22]. Advanced maternal age also seems to serve as a risk factor, with pregnancy loss rates rising rapidly after the age of 30 [1,23].

Amongst women with anatomical uterine anomalies, the highest incidence of recurrent pregnancy loss occurred in those diagnosed with septate uteri [24]. Elevated thyroid-stimulating hormone (TSH) levels are a well-documented risk factor for pregnancy loss, as well as abnormal fetal development [25,26]. Given progesterone’s role in implantation and early fetal development, any condition associated with progesterone deficiency may be associated with recurrent pregnancy loss [24,25]. Chronic endometritis is also linked to RPL, with evidence showing that the uterine endometrium microbiome can predict pregnancy success rates [27,28]. Factor V Leiden point mutation (G1691A) is the most common form of genetic thrombophilia and is correlated with recurrent pregnancy loss [29].

Genetic factors linked to RPL include parental karyotype abnormalities (translocations, inversions, copy number variants) [2,30], parental chromosomal polymorphisms [31,32,33,34], an increase in sperm aneuploidy [35,36,37], fetal microchimerism [38], severe skewing of X chromosome inactivation [39], and various gene polymorphisms [40,41,42,43,44,45,46].

A recent GWAS meta-analysis identified three distinct genome-wide significant loci for recurrent pregnancy loss, located on chromosomes 9, 11, and 21, containing potential candidate genes (*FGF9*, *TLE1*, *TLE4*, *E2F8*, *SIK1*) linked to placental biology in patients of European ancestry [47].

A large-scale review managed to identify a modest degree of association between idiopathic RPL and 21 parental gene variants involved in the immune response, coagulation, metabolism and angiogenesis, thus emphasizing the need for more gene studies [48].

Despite significant advances in testing, the underlying etiology of RPL remains elusive in a substantial number of cases, requiring complex management protocols [49].

Our study aims to explore the role of conventional karyotyping in couples with RPL in a southwestern Romanian population. A stratified analysis based on the frequency and type of chromosomal abnormalities was also performed.

## 2. Materials and Methods

### 2.1. Subjects

The peripheral blood samples included in this study were referred to the Human Genomics Laboratory of the University of Medicine and Pharmacy in Craiova, Romania for conventional cytogenetic analysis between January 2013 and December 2023, by the Outpatient Medical Genetics Clinic of the Emergency Clinical County Hospital of Craiova. Data concerning reproductive history were obtained in order to include all cases with a positive history of RPL. The study was conducted in accordance with the Declaration of Helsinki and approved by the Ethics Committee of the University of Medicine and Pharmacy of Craiova, Romania (no. 44/24 March 2022).

### 2.2. Conventional Cytogenetic Analysis

Chromosome analysis was performed using whole human peripheral blood samples collected in sterile vacutainer tubes containing sodium heparin. Following delivery to the laboratory, two separate lymphocyte cultures were immediately established for each patient, using 0.5 mL of whole blood and 10 mL of PB-MAX™ Karyotyping Medium (Gibco Invitrogen, Waltham, MA, USA). Cell cultures were incubated for 72 h in T-25 cell culture flasks at 37 °C. The cells were then arrested in metaphase with colcemid, processed using hypotonic potassium chloride solution and methanol–acetic acid 3:1 fixative, and spread on microscope slides, which were subsequently Giemsa-trypsin banded, according to standard protocols. At least 20 metaphases were analyzed and karyotyped for each sample (Ikaros v5.4, Metasystems, Altlussheim, Germany). The karyotypes were reported according to ISCN 2013, 2016, and 2020. Data were analyzed by using SPSS Statistics for Windows, Version 22.0 (IBM SPSS Statistics for Windows, Version 22.0. Armonk, NY, USA: IBM Corp).

## 3. Results

A total of 213 couples (426 individuals) with a history of RPL were enrolled in this retrospective study. The age of the female study participants ranged between 21 and 44 (a mean age of 33.8 years old), while their male counterparts were aged between 25 and 52 (a mean age of 35.6 years old), with pregnancy losses occurring between 5 and 16 weeks of gestation. Out of 426 patients who underwent a conventional G-banded chromosome analysis, 410 (96.25%) had a normal karyotype, and 16 had either numerical or structural chromosome abnormalities (3.75%) (Table 1).

**Figure 1:**
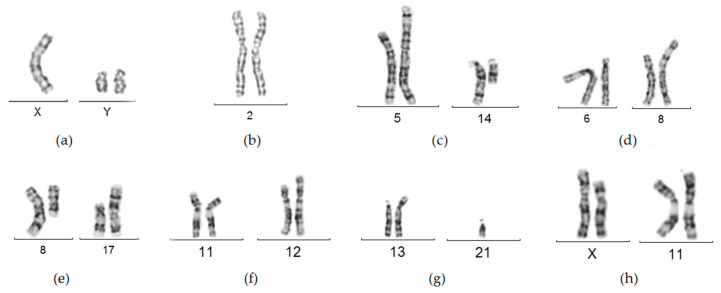
Partial karyotypes of some chromosome abnormalities detected in RPL couples: (**a**) XXY; (**b**) inv(2)(p11.2q13); (**c**) t(5;14)(p12;q11); (**d**) t(6;8)(p12;q13); (**e**) t(8;17)(q12;p12); (**f**) t(11;12)(p14;p13); (**g**) der(13;21)(q10;q10); (**h**) t(X;11)(p11.2;p13).

All structural chromosomal rearrangements (3.52% overall) were balanced—either a balanced translocation involving mainly chromosomes 8, 11, 14, and 21 (14 cases) or an inversion with inv(2) being the only one detected. (Table 2 and Figure 1). The only numerical anomaly detected was in a 47,XYY male with a normal 46,XX female partner.

We also observed various chromosomal polymorphisms which included length variations in the heterochromatic regions of chromosomes 1, 9, 16, and Y, variations in the size of satellite and/or stalk regions of chromosomes 13–15 and 21, 22, as well as pericentric inversions of chromosomes 1 and 9. The most common single polymorphism identified was inv(9) in both men and women (11.5% and 15.4%, respectively), followed by 1qh+ and 9qh+ in women (7.7% and 3.8%, respectively), and Yqh+ and 9qh+ in men (9.6% and 4.8%, respectively). Table 3 compiles all chromosomal polymorphisms detected in the participants.

## 4. Discussion

We explored the value of conventional karyotyping in couples with RPL and found that the incidence of structural chromosomal abnormalities was 3.52% amongst individuals meeting RPL criteria. However, if we were to exclude couples in whom karyotyping was performed due to POC/chorionic villi/amniotic fluid samples with unbalanced structural abnormalities, the abnormality rate is much lower (0.94% of individuals). Similar results (2.9%) were reported in a large meta-analysis [50], as well as different studies in Israel (2.52%) [51], Iran (3.15%) [52], and Saudi Arabia (2.56%) [53]. Nonetheless, other studies reported either a higher or lower percentage of abnormal cases; this rate is quite variable and can be attributed to various factors, such as sample size, case selection, ethnic homogeneity, the inclusion of chromosomal polymorphisms, etc. For instance, a higher incidence was observed in Japanese (7.8% of couples) [54], Indian (8.7% couples) [55], Egyptian (6.25% of individuals) [56], and Tunisian (8.65% of couples) [57] cohorts, while a lower incidence was found in Canadian (2.7% of couples) [58], Dutch (3.2% of couples) [59], and Chinese (1.77% of couples) [60] populations.

A large retrospective study conducted in the United Kingdom found an incidence of balanced chromosomal abnormalities amongst couples that had experienced a miscarriage of only 1.9% [61].

Numerical chromosome abnormalities can be present as sex chromosome aneuploidies in RPL couples, occurring at a low rate of around 0.15% of tested individuals [62,63]. Our study includes a single 47,XYY male (0.23%); males with XYY syndrome have been shown to be more prevalent in infertile populations [64].

We found that reciprocal (80%) and Robertsonian (13.3%) translocations were the most frequent structural abnormalities detected, and they involved all chromosomes except for chromosomes 10, 16, 19, 20, and Y. In our study, balanced translocations seemed to occur more frequently in women rather than men (F:M ratio of 2:1), in line with most studies [65,66,67]. Women appear to be more likely to be carriers of a translocation (either reciprocal or Robertsonian) or an inversion, with one explanation being that, in humans, structural chromosome abnormalities usually compatible with fertility in females may be associated with sterility in males [68].

Analyzing data from six centers for clinical genetics in the Netherlands, Franssen et al. recorded 278 structural chromosome abnormalities, consisting of 177 reciprocal translocations (64%), 43 Robertsonian translocations (15%), 21 pericentric inversions (8%), 21 paracentric inversions (8%), and 16 other structural chromosome abnormalities (6%). The carrier sex ratio was skewed, with 176 (63%) of carriers being women [59]. Another Dutch nested case–control study, which included 1324 couples with a history of RPL, found a structural chromosome rearrangement carrier rate of 2.5%: eighteen female and eight male carriers of a reciprocal translocation (63%), three carriers (7%) of a Robertsonian translocation, and nine carriers (22%) of an inversion [63]. Balanced reciprocal translocations were also the most frequent chromosome abnormalities in couples investigated for RPL, followed by Robertsonian translocations, while a 47,XXY karyotype was found in seven out of 16,692 men in a large Canadian cohort that included 44,398 subjects [68].

A particularly high percentage (29.4%) of inversions was noted in an Indian cohort, but this study also included heteromorphic variants (the pericentric inversions of chromosomes 9 and Y) in the structural abnormality subgroup [55].

It is still generally accepted that there is an increased risk of abnormal viable offspring for carriers of a balanced chromosomal abnormality, with the possibility of a POC karyotype of being either balanced (a normal or balanced carrier) or unbalanced, leading to miscarriage, stillbirth, or abnormal phenotypes. Out of the eleven cases of abnormal POC/chorionic villi/amniotic fluid sample karyotypes included in our study, four showed no fetal anomalies on routine ultrasound examinations, in line with balanced karyotype findings, and with no probable genetic cause—46,XX,inv(2)(p11.2q13); 46,XX,t(1;15)(p36.3;q26.1); 46,X,t(X;11)(p11.2;p13); 46,XY,t(5;14)(p12;q11). Fetal ultrasound anomalies were observed in five cases: hydrops fetalis and cystic hygroma—45,XX,der(9)t(9;21)(p24.3;q22.1),-21; nuchal edema, ventriculomegaly, non-visualization of the fetal cavum septi pellucidi, unilateral cleft lip and palate, agenesis of the fetal portal system, dextrocardia, agenesis of the ductus venosus, non-visualization of the stomach, and clenched hands—46,XX,der(12)t(11;12)(p11;q24); lateral ventricular asymmetry, single umbilical artery, and paracentral umbilical cord insertion—46,XX,del(18)(q21.3q23); unilateral cleft lip and palate—46,XX,t(8;17)(q12;p12); cystic hygroma, absent nasal bone, reversed A-wave in the ductus venosus, and tricuspid regurgitation—46,XY,der(13;21)(q10;q10),+21. We had no fetal ultrasound data for the remaining cases—46,XY,der(15)t(3;15)(q21;q15) and 46,XX,del(11)(p14)—with losses having occurred in early pregnancy (6–9 weeks).

Previous guidelines regarding the management of RPL included parental karyotyping after two or three miscarriages [69,70,71]. More evidence seems to suggest that RPL couples have a high probability of producing normal offspring, despite a higher risk of miscarriage [59,61,63,72,73]. In our study, a single child subsequently diagnosed with Down syndrome was born 15 years ago from a female balanced translocation carrier—46,XX,t(9;21)(p24.3;q22.1)—with no ultrasound and biochemical screening performed during pregnancy. At present, with prenatal screening methods such as NIPT and high-resolution fetal ultrasound becoming increasingly available, this case would have most likely been diagnosed prenatally.

In light of these findings, recent ESHRE and RCOG guidelines reconsidered the value of parental chromosomal testing, since there is a negligible chance of live birth with an unbalanced chromosome abnormality for the unselected RPL population [2,4]. The ESHRE suggests using chromosomal microarray platforms (CMA) for POC genetic testing based on its maternal contamination reducing effect [4], but since CMA is unable to detect balanced chromosome abnormalities that could lead to unbalanced POCs, this method is less valuable when it comes to evaluating RPL couples. Recurrent miscarriages are highly emotionally distressing events; however, karyotyping all individuals meeting RPL criteria is not feasible from an economic standpoint [61,74,75], with the guidelines recommending paternal karyotyping after individual risk assessment, such as a previous live birth with congenital anomalies and/or unbalanced chromosomes or if a structural abnormality is identified in products of conception (POCs) or if there is unsuccessful or no pregnancy tissue available for testing [2,4]. Given the fact that the incidence of chromosomal abnormalities in RPL couples is not considerably higher than that of the general population, karyotyping couples is not only a less valuable, but also a more time-consuming and cost-inefficient diagnostic test in the absence of suggestive POC karyotype alterations.

Genetic counseling and prenatal testing, such as chorionic villus sampling (CVS) or amniocentesis, are used to assess future pregnancies in RPL couples consisting of at least one partner carrying a balanced chromosomal rearrangement. These couples may also constitute potential candidates for in vitro fertilization (IVF) and preimplantation genetic testing (PGT), particularly when it comes to sperm aneuploidy [76]. However, this does not constitute a routine recommendation.

We found that 104 out of 426 participants (24.4%) had one or several chromosome heteromorphic variants. The issue of these variants and their role in infertility remains controversial, with some studies providing a link between meiotic dysfunction and heterochromatin variability [77,78,79,80]. In the absence of any recent large-scale studies, the overall consensus lies with Bobrow’s review of the literature [81], with the impact of chromosomal heteromorphic variants on reproduction being largely considered benign.

Our study is limited by the small sample size. Clinical data regarding the pregnancy losses of female RPL patients were obtained via a simple questionnaire, being scarce and subjective, but nonetheless providing the minimal diagnostic requirements for RPL. A subsequent larger study is required in order to assess statistically significant correlations between the karyotype, age and reproductive background of RPL couples and their respective number of miscarriages, POC karyotypes, and future pregnancy outcomes.

## 5. Conclusions

Our study provides evidence that, in unselected RPL couples, the purpose of karyotyping is mainly to provide valuable peace of mind to the great majority of couples that they have a normal karyotype, rather than to detect translocation carriers and prevent abnormal live births. Following the identification of POC chromosomal abnormalities, paternal karyotyping can offer reassurance by suggesting some potential underlying genetic causes of the miscarriages and thus ultimately improving pregnancy outcomes for couples experiencing RPL. However, in the absence of a positive history, blindly karyotyping couples may prove too expensive and labor intensive, while also providing no information on fertility status or live birth rates.

## Figures and Tables

**Table 1:** Karyotype breakdown of individuals in couples with RPL.

Karyotype			Females *n* (% t)	Males *n* (% t)	Total *n* (%)
Normal	46,XX or 46,XY		155 (36.4%)	151 (35.45%)	306 (71.85%)
	Chromosomal polymorphisms		48 (11.25%)	56 (13.15%)	104 (24.36%)
Abnormal	Numerical abnormalities	Sex trisomy	-	1 (0.25%)	1 (0.25%)
	Structural abnormalities	Translocations	10 (2.35%)	4 (0.9%)	14 (3.3%)
		Inversions	-	1 (0.25%)	1 (0.25%)
Total			213 (50%)	213 (50%)	426 (100%)

**Table 2:** Structural chromosome abnormalities in RPL couples.

Type	*n* (%)		Karyotype	Product of Conception/Chorionic villi/Amniotic Fluid Sample
Inversions	1 (6.7%)	Present in male	46,XY,inv(2)(p11.2q13)	46,XX,inv(2)(p11.2q13)
Reciprocal translocations	8 (53.3%)	Present in female	46,XX,t(1;15)(p36.3;q26.1)	46,XX,t(1;15)(p36.3;q26.1)
			46,XX,t(2;4)(q22;q33)	None
			46,XX,t(3;15)(q21;q15) 46,XX,t(7;22)(p21;q13) 46,XX,t(9;21)(p24.3;q22.1)	46,XY,der(15)t(3;15)(q21;q15)
				None
				45,XX,der(9)t(9;21)(p24.3;q22.1),−21
			46,XX,t(11;12)(p11;q24)	46,XX,der(12)t(11;12)(p11;q24)
			46,XX,t(17;18)(p13;q21.3)	46,XX,del(18)(q21.3q23)
			46,X,t(X;11)(p11.2;p13)	46,X,t(X;11)(p11.2;p13)
	4 (26.7%)	Present in male	46,XY,t(5;14)(p12;q11)	46,XY,t(5;14)(p12;q11)
			46,XY,t(6;8)(p12;q13)	None
			46,XY,t(8;17)(q12;p12)	46,XX,t(8;17)(q12;p12)
			46,XY,t(11;12)(p14;p13)	46,XX,del(11)(p14)
Robertsonian translocations	2 (13.3%)	Present in female	45,XX,der(13;21)(q10;q10)	46,XY,der(13;21)(q10;q10),+21
			45,XX,der(14;21)(q10;q10)	None
Total	15 (100%)			

**Table 3:** Chromosomal polymorphisms (CPM).

Chromosome	Chromosomal Polymorphism (CPM)	Female to Male Ratio	*n*	% of Detected CPMs
Non-acrocentric	1qh+	4:0	10	9.6
	9qh+	4:5	9	8.65
	9qh−	-	2	1.95
	16qh+	2:3	5	4.8
	inv(1)	1:1	2	1.95
	inv(9)	4:3	28	26.9
Acrocentric	13ps+	-	1	0.95
	13pstk+	1:1	2	1.95
	14ps+	-	1	0.95
	14pstk+	-	1	0.95
	15ps+	1:3	4	3.85
	15pstk+	-	2	1.95
	21ps+	1:2	6	5.8
	21pstk+	1:3	4	3.85
	22ps+	-	2	1.95
	22pstkpstk	-	1	0.95
Y chromosome	Yqh+	-	10	9.6
	Yqh−	-	2	1.95
Multiple occurring variants	9qh+,21ps+	-	2	1.95
	inv(9),9qh+	-	1	0.95
	inv(9),21ps+	-	1	0.95
	14pstk+ps+	-	1	0.95
	1qh+,9qh+	-	1	0.95
	1qh+,16qh+	-	1	0.95
	9qh+,15pstk+	-	1	0.95
	9qh-,21ps+	-	1	0.95
	14ps+,22ps+	-	1	0.95
	Yqh+,21pstk+	-	1	0.95
	Yqh-,21pstk+	-	1	0.95
Total			104	100

## Data Availability

All data presented here are available from the authors, upon reasonable request.
